# Report of Committee on Diseases*Reported at the 29th Annual Meeting of the United States Veterinary Medical Association.

**Published:** 1892-12

**Authors:** A. W. Clement


### REPORT OF COMMITTEE ON DISEASES.*

By A. W. Clement, V.S.

*Mr. Chairman and Gentlemen:*—So many are engaged at present in the investigation of diseases, and the literature upon the subject is so very voluminous, that a mere repetition of the conclusions arrived at would make of itself a report too extensive for this occasion. I have, therefore, decided to confine my remarks to the question of immunity and cure of infectious diseases, inasmuch as important discoveries have been made in this direction, and the question has equally a scientific and practical value.

We seem to be at the beginning of a new era in therapeutics, based upon a clearer understanding of the etiology of the infectious diseases. Specific remedies have long been sought, but it is only recently that we have learned where to look for them. We have only passed over the threshold of the new knowledge. The practical application of the scientific truths discovered is not in all respects clear, but we can confidently predict that these truths are going to lead to important advancement in the prevention and treatment of infectious diseases. It is a matter of just pride to us that the first demonstration that immunity can be produced by the chemical products of bacteria, was made in this country and by one of our own members, Dr. D. E. Salmon, chief of the Bureau of Animal Industry. This discovery was made in the case of hog cholera, published in a paper read before the Biological Society, at Washington, in the beginning of 1886.

A great deal has been done in this line which is quite suggestive of what we may expect in the near future. You are all aware that infectious diseases are produced by the introduction into the system of specific micro-organisms. That these micro-organisms produce disease in two ways: 1. By toxic effect, in which case the bacteria, growing locally, give rise to constitutional symptoms by an absorption of their poisonous products, e. g., "Tetanus," "Diphtheria," "Asiatic Cholera," etc. Secondly, by a septicæmia in which the organisms grow rapidly throughout the whole body, and in which poisonous products are formed, but in less concentration.

It is well known that not only must we have the disease-producing agent, but that the animal body which receives this agent,

*Reported at the 29th Annual Meeting of the United States Veterinary Medical Association.

must be, at the time, in a condition capable of permitting the growth of the agent, and also incapable of neutralizing the poison. If then we can bring about such a condition that the invading micro-organism will not grow, we have the condition of immunity. We can also prevent infection by rendering the body proof against the poison. Recent researches appear to show that more is to be expected from means of rendering the body poison-proof than from means of directly killing the bacteria. If we are able to neutralize the poison produced by bacteria, we deprive them of their principal weapon of attack and reduce them to the condition of harmless saprophytes.

One of the most important discoveries is that the natural cure of an infectious disease is due to the formation of an immunity substance in the body, this substance being a direct antidote to the poison. If we can introduce directly this immunity substance, or hasten its development, we can cure the disease. In other words, the new specific remedies are such as accomplish these ends.

Infectious diseases themselves confer immunity against a second attack, for a greater or less length of time, by the formation of an immunity substance which acts as an antidote against a new invasion of the same organism. An attack of small pox confers immunity against further attack, as a rule, during the patient's lifetime. An attack of croupous pneumonia confers immunity in human beings only for a short time. It is upon a knowledge of these facts that the system of experimental therapeutics on the line of immunity has been inaugurated.

Much has been accomplished in the laboratory. Much remains to be done before it can be put to practical use. I do not propose to review the whole literature upon this question, but to confine my remarks, principally, to a brief review of what has been done in some of the diseases which are of practical interest to us, as veterinarians.

I will speak first of "Tetanus." The time has long since passed when the old idea that "Tetanus" is a purely nervous disease, produced by injury to a nerve, or from an unknown cause giving rise, in the first instance, to the term "traumatic," and in the second instance to the term "idiopathic tetanus." Shortly before the discovery of the tetanus bacillus it was shown by Carle and Rattoni that animals inoculated with the pus from individuals affected with "tetanus," developed the same disease, thus proving that it was due to infection.

In 1884 Nicolaier, at the Hygienic Institute, at Göttingen, produced the disease in rabbits by the subcutaneous inoculation of garden earth and discovered the specific tetanus bacillus.

In 1886 Rosenbach demonstrated the presence of the same organism, though mixed with others, in the pus at the seat of inoculation in human beings. Great difficulties were encountered in obtaining the organism in a pure state on account of its being mixed with others, and on account of its being strictly anærobic, so the etiological significance of the organism was not definitely established until Kitasato obtained pure cultures, which he did by an ingenious process in 1889, depending on the fact that the other bacteria which were present were killed by exposure to a temperature of 80 degrees centigrade for a period of from three-quarters of an hour to an hour, while the tetanus bacilli survived this temperature for this length of time. These bacilli planted in liquefied agar, preferably containing about two per cent. of grape sugar. through which liquefied agar hydrogen gas was passed to displace the oxygen, grew in pure cultures.

The bacilli of "tetanus" are straight rods, or thread may be formed. These bacilli form spores especially rapidly at high temperature. The spores are round and situated at the end of the rods which gives a drum-stick appearance to the organism. They possess distinct but sluggish independent motility. After the spores are formed the bacilli are non motile.

The spore material will withstand complete drying for many months. They will, in a moist condition, survive a temperature of 80 degrees centigrade for an hour. They are not killed in five per cent. carbolic acid solution after an exposure of ten hours, but are killed after fifteen hours. They are killed in three hours in a one to one thousand sublimate solution. They liquefy gelatine slowly with a feeble gas production. The growth in solid cultures may be compared to the appearance of a brush such as is used for cleaning lamp chimneys. The bacillus grows best at the body temperature, but will grow slowly at 18 to 20 degrees centigrade. There is no growth under 14 degrees centigrade.

There is no organism to which a large number of different species of animals are susceptible. Chickens are the only animals positively known to be naturally immune, although not all animals have been tested.

The bacilli are toxic in their effects and produce death by the poison which they form. In animals inoculated with a pure culture of tetanus, the bacilli develop locally but do not invade the blood. They are found only at the seat of inoculatlon. The tetanic symptoms appear first near the part of the body inoculated. For example, if the inoculation is made in the left hind leg the muscles in that leg will be first affected. If the inoculation be in the neck, the cervical muscles are first stiffened, The symptoms gradually become more general, and finally we have convulsions which are excited especially by external influences. At the autopsy of animals inoculated with a pure culture, we find no marked local reaction. There is hyperæmia but no suppuration at the point of inoculation.

In natural infection with "tetanus" it is to be remembered that other organisms are present besides the tetanus bacilli, and these other organisms may be such as cause suppuration, gangrene, etc.

In Kitasato's experiments with pure cultures the curious fact was noted that the bacilli die before the animal dies. Neither at the seat of inoculation, or elsewhere, can the bacilli be found. This will explain why it is possible to miss the bacilli at the point of inoculation in animals dead of natural infection.

One can form some idea of the intensity of the poison when he bears in mind that experiments on mice have been made with a view of stopping the action of the bacilli by extirpation of the inoculated area soon after the inoculation has been made. One hour after the inoculation a wide area has been cut out at the seat of inoculation and the surface burned. This failed to check the progress of the infection, showing that already enough poison had been produced and absorbed to kill. This applies to mice inoculated with pure cultures, but the conclusion may be drawn that before any symptoms have made their appearance, the removal of large areas containing all of the bacilli will not necessarily prevent the development of the disease.

Subcutaneous inoculations of mice, with pure cultures, develop symptoms in twenty-four hours and cause death in two or three days.

Mention of the manner in which the tetanus organism kills. leads us to a study of the chemistry of the organism.

Brieger in 1887 endeavored to obtain the poison products. He found crystallizable alkaloidal substances of the class called "Ptomaines." He designated them as "Tetanine" and "Tetanotoxine."

"Tetanine" produces symptoms something like those caused by strychnine, and these he supposed to be the specific poisons of the disease. This has since been found to be incorrect, as a far more deadly poison was subsequently demonstrated by Brieger and Frankel, which belongs to a totally different class of substances, namely, the "toxic albumens," to which class we know now that most of the specific chemical poisons of bacteria belong, and not to the "Ptomaine" class, as formerly supposed

This "toxic albumen" of the tetanus organism is contained in the filtrate obtained by passing a liquid culture of the tetanus bacillus through a Pasteur filter, precipitating with alcohol and dissolving with water.

Inoculations with this bacteria-free filtrate into animals produce all of the symptoms of "tetanus" in a length of time proportionate to the size of the dose. The symptoms may appear in from seven hours to three days. Blood from animals sick with the disease will produce "tetanus" in one generation following.

From a rapid review of the description and history of the organism with its chemistry, let us see what has been done in the production of immunity, both preventive and curative.

Kitasato first succeeded in rendering rabbits immune against "tetanus" by injecting the filtrate of a bouillon culture of tetanus bacilli, and following the injection immediately by the injection of the trichloride of iodine, and repeating the trichloride of iodine injection at intervals of twenty-four hours for several injections. These experiments were successful, however, in only forty per cent. of the rabbits treated, and it had no effect on mice and guinea pigs. The blood of the immunized rabbits, it was found, would confer immunity on other animals, including mice and guinea pigs, and would cure the disease when injected into infected animals after the symptoms had developed.

It was found, at the same time, that the blood of chickens, which are naturally immune against the disease, when injected into rabbits and mice, would not confer immunity against "tetanus." This may be laid down as a rule: That only animals rendered immune artificially can confer immunity to other animals. The property conveyed to the blood of an animal when it is immunized is the power of destroying the poison of the disease, but only of the poison of the dicease against which it has been rendered immune. Thus, an animal which has been rendered immune against "tetanus" is not protected against any other disease, such as chicken cholera, diphtheria, etc.

In addition to the method of immunization before mentioned, the culture itself may be treated with the trichloride of iodine, which makes an immunizing fluid against "tetanus" even for mice, which do not respond to the first method.

Horses and sheep have been rendered immune by Behring and Schutz, by treatment with the tetanus bouillon filtrate, containing carbolic acid and trichloride of iodine.

The method of immunizing with trichloride of iodine has a serious practical objection, from the fact that the drug itself has very caustic effects and that there are more failures than successes by this method.

Brieger, Kitasato and Wasserman have recently found the most successful agent of vaccination against "tetanus" to be the following: A mixture of a twenty-four hour culture of the tetanus bacillus in neutral peptone bouillon, with the extract of the thymus gland of a calf, in proportion of one part of the culture to two parts of the extract from the thymus gland. This mixture, allowed to stand for several days in the cold, has been found to be an excellent vaccine, with which all the rabbits experimented upon were rendered immune, as was also a goat. The juice of the thymus gland has the specific power of neutralizing the poison of "tetanus." By this new method of vaccination we are able to produce immunity in animals positively and readily.

The blood of the animals, for instance, rabbits, goats, horses, etc., contains a new substance which acts as a direct antidote to the tetanic poison. This is proven by mixing poisonous bouillon culture of the tetanus bacillus with this immunized blood, and finding that thereby these cultures are deprived of their toxic properties. Also and more to our purpose, the same important fact can be demonstrated by injecting the blood serum of an immunized animal into a second animal, and in this way a second animal may itself be rendered immune against the disease. We must, however, as Ehrlich has shown, distinguish between immunity conferred in this way and that produced by vaccination.

In the first case we introduce directly the already formed immunity substance. Immunity is produced at once or in a few hours, but the degree of immunity is only a fraction of that of the original animal. Whereas, when we produce immunity by the vaccine substance, we must wait for several days for the formation of the protective immunity material, but in compensation we can attain a higher degree of immunity in this way than by the use of the blood serum. This matter of the degree of immunity is important. Ehrlich proposes to designate in figures the degree by determining how large a fatal dose the vaccinated animal will stand. Thus the degree of immunity may be 2–20–500–1000, etc., in other words the animal will stand 2–20–500–1000, etc., times the fatal dose.

Not only can we use the blood of a vaccinated animal to produce quick immunity, although not of high degree, in a second animal, but we can actually prevent the development of the disease after inoculation with the specific organism ; even more, we can cure the disease after it has developed, if it is not too far advanced. In other words, the blood serum of an immunized animal possesses curative properties.

This blood serum therapy, as it is called, is now a demonstrated scientific fact, whatever may be the fate of its practical application. It has been applied already successfully in the treatment of "tetanus" in human beings. These patients were cured of "tetanus," which, as is well known, is very fatal, by using the immunity substance obtained from the blood of rabbits rendered artificially immune.

The degree of curative power possessed by the blood serum depends upon the degree of immunity attained. According to Kitasato, the more susceptible an animal to the disease, and the higher the degree of immunity produced artificially, the greater the curative power of the blood serum. He believes that the horse, rendered highly immune by vaccination, will furnish the most healing blood serum.

There are some practical difficulties in the application of blood serum therapy, which at present forbid its general application. First. The blood serum of one species of animal is more or less injurious to another species. This consideration is not without force if we have to use large quantities of the blood serum, and it is probable that in endeavoring to cure such large animals as the horse and man, it will be necessary to use such large amounts of the curative serum, that the application would be virtually a transfusion of blood. If, however, we are able to obtain the blood from an immunized animal of the same species, this danger would be greatly reduced. Secondly. It is not easy to obtain and preserve the blood serum in an aseptic condition. Thirdly. There are practical difficulties in obtaining at the right moment vaccinated animals.

A large part of these difficulties would be overcome if we were able to isolate, as a distinct permanent chemical substance, the curative substance from the blood. Efforts in this direction are only partly successful thus far, but we may hope that they will be eventually successful.

In the meantime, experiments have been made to obtain directly from cultures curative substances. You are familiar with the first step in this direction, viz.: the introduction of "tuberculin" by Koch.

The expectations originally held have unquestionably been disappointing as regards the healing power of this substance. Its diagnostic value has been proven. In this connection I need only refer to the valuable and accurate researches of the "Tuberculosis Commission of the University of Pennsylvania," of which Dr. Zuill is chairman.

The belief is entertained by Buchner and others that Koch's "tuberculin" is essentially a solution of the protein constituents of the tubercle bacillus. We distinguish the specific poison produced by bacilli from their bacteria-protein from other kinds of bacteria.

It has been found that the concentrated bouillon culture of the diplococcus pneumoniæ, heated to 60 degrees C., are deprived of poisonous properties, but are capable of conferring a high degree of immunity, and the culture material thus treated has been successfully used in treating pneumococcus infection, both in animals and in human beings, so that we have here an instance in which the culture material can be used as a therapeutic agent. Such material is much more easily prepared and manipulated than the curative blood serum, and we may hope that we shall come into the possession of valuable therapeutic agents obtained directly from cultures.

The general principles which have been described regarding the production of immunity and the cure of "tetanus" are applicable to many other diseases, such as swine erysipelas, or "Rothlauf," pneumococcus infection, diphtheria, etc.

The interesting discovery has been made by Tizzoni and Centanni that the blood serum of rabbits, rendered immune against rabies by Pasteur's vaccination, is a direct antidote to the specific poison of rabies, depriving virulent spinal cords from rabid animals of all toxic property. This blood serum is even capable of curing rabies in inoculated rabbits after symptoms have appeared. This discovery is a welcome addition to Pasteur's work.

"Glanders" has received considerable attention lately, with the result that a substance of great diagnostic value, called "Mallein," has been discovered. This substance has undisputed value as a diagnostic agent reacting in animals the subject of "glanders," and having no effect on healthy animals. I refer you for details to Dr. Pearson's writings upon the subject in the *American Veterinary*

*Review* and the JOURNAL OF COMPARATIVE MEDICINE AND VETERINARY ARCHIVES.

In conclusion, your committee takes great pride in recording the fact that such an active part is being taken by our American veterinarians in the scientific and practical investigations of the diseases which affect our domesticated animals.

